# The Mechanism of Lavender Essential Oil in the Treatment of Acute Colitis Based on “Quantity–Effect” Weight Coefficient Network Pharmacology

**DOI:** 10.3389/fphar.2021.644140

**Published:** 2021-04-22

**Authors:** Yao Wang, Junbo Zou, Yanzhuo Jia, Xiaofei Zhang, Changli Wang, Yajun Shi, Dongyan Guo, Zhenfeng Wu, Fang Wang

**Affiliations:** ^1^ Department of Pharmaceutics, College of Pharmacy, Shaanxi University of Chinese Medicine, Xianyang, China; ^2^ Department of Pharmaceutics, College of Pharmacy, the Key Laboratory of Basic and New Drug Resea Rchof Traditional Chinese Medicine, Shaanxi University of Chinese Medicine, Xianyang, China; ^3^ Department of Pharmaceutics, Jiangxi University of Traditional Chinese Medicine, Nanchang, China

**Keywords:** lavender essential oil, acute colitis, network pharmacology, bioinformatics, signal pathway

## Abstract

This study aimed to introduce a new weight coefficient combined with network pharmacology to predict the potential active components, action targets, and signal pathways of lavender essential oil and to investigate the therapeutic effect of lavender essential oil on colitis through animal experiments. The component targets of lavender essential oil were mined from the Pubchem and SwissTargetPrediction databases, and the relative content of lavender essential oil was compared with OB (oral bioavailability) to establish a “quantity–effect” weight coefficient. Online databases such as GeneCards and String were used to construct a “lavender essential oil compound target disease target” network to extract the key targets of core compounds acting on diseases. The clusterProfiler package in R language programming of Rstudio software was used to analyze the enrichment of the related targets by Gene Ontology and the Kyoto Encyclopedia of Genes and Genomes (KEGG), and the enriched pathways were reordered according to the “quantity–effect” weight coefficient of the targets they participated in. Following up on the findings, the pharmacodynamic test showed that, after injecting lavender essential oil into mice, the levels of inflammatory cytokines including EGFR, TNF-α, and IFN-γ in serum and colon tissue decreased, and lavender essential oil could mediate Th17 cell differentiation by reducing dextran sulfate sodium (DSS)-induced ulcerative colitis (UC) colonic mucosal damage. The results indicated that lavender essential oil can alleviate DSS-induced colonic mucosal injury in ulcerative Colitis mice. Based on the network pharmacology of the “quantity–effect” weight coefficient, this study indicated that lavender essential oil can regulate the level of inflammatory factors, inhibit inflammatory reactions through a multicomponent and multitarget strategy, and ultimately alleviate the colonic mucosal injury of UC mice. Through the weight coefficient network pharmacology mining, it was concluded that the Th17 cell differentiation, PI3K-Akt signaling pathway, and Th1 and Th2 cell differentiation of lavender essential oil in the treatment of UC may be the key pathway for the treatment of the disease. Through the establishment of a weight coefficient combined with network pharmacology and the combination of dose and effect, it shows that network pharmacology may provide a better basis for the treatment of disease mechanism.

## Introduction

*Lavandula aspic* L. is a strong aromatic shrub of Labiaceae. Lavender essential oil has a long history in treating diseases. The nuances of different lavender smells are due to the difference in the composition and content of essential oils. This difference can directly affect the function or pharmacological action of essential oils (Arantes et al., 2016). At present, lavender essential oils are still as widely used and tested as they were last century. It has antibacterial, relaxing, and soothing effects, as well as significant effects on burns and insect bites. Colitis, a recurrent gastrointestinal disease mainly involving the colorectal mucosa and submucosa, is difficult to treat (Baumgart and Sandborn, 2007; Kaser, Zeissig, and Blumberg, 2010). In recent years, the global incidence rate has increased and affected patients have tended to be younger (Ng et al., 2017). Modern medicine has a variety of treatments for different grades of ulcerative colitis (UC) (Cao 2013), but these also have severe adverse effects in addition to curative effects, such as opportunistic infections, bone marrow toxicity, and tumor induction (Guo et al., 2017).

Few experimental studies have been conducted on the molecular mechanism of lavender essential oil in the treatment of UC. Baker et al. (2012) reduced the destructive proinflammatory response of lavender essential oil by extracting medicinal lavender essential oil to regulate intestinal flora, which showed that it played an important role in promoting the balance of gastrointestinal immunity so as to serve as a potential treatment for colitis. However, its specific mode of action was not elucidated.

Network pharmacology has a wide range of applications in the study of the material basis and mechanism of drug efficacy, the compatibility of compounds, and the new drug research (Tang et al., 2015). Although it provides convenience, it also has some shortcomings. Specifically, network pharmacology research relies more on public databases, so the accuracy, reliability, and completeness of the data have a great impact on the prediction results (Aminu et al., 2017). Its data information needs to be further supplemented and verified. Moreover, the same drug with a different harvest time, growth environment, or other conditions can affect the differences in the composition of medicinal materials. Second, most studies of network pharmacology are based on the qualitative analysis of the "component target disease" network, but they ignore the influence of the content of each component. As such, there is an urgent need to design corresponding network algorithms to quantitatively evaluate how drugs act.

However, the key factor of component content is often ignored in current network pharmacology research. As the content and concentration of drugs are critical to the efficacy, only when a certain amount of drugs reach the target position can the drug play its role. In other words, all components are treated equally, which is unreasonable. Second, OB and DL are generally used as screening conditions in current network pharmacology research in traditional Chinese medicine to determine the screening basis of effective components. Only components that can be well absorbed after oral administration can become effective components. Although components with high content but poor absorption can be avoided as effective components, the influence of component content on efficacy is ignored. This often leads to drugs with a low content of components being considered effective components, while those with a high content of components are considered ineffective components, which makes more network pharmacology lose its original significance.

**FIGURE 1:**
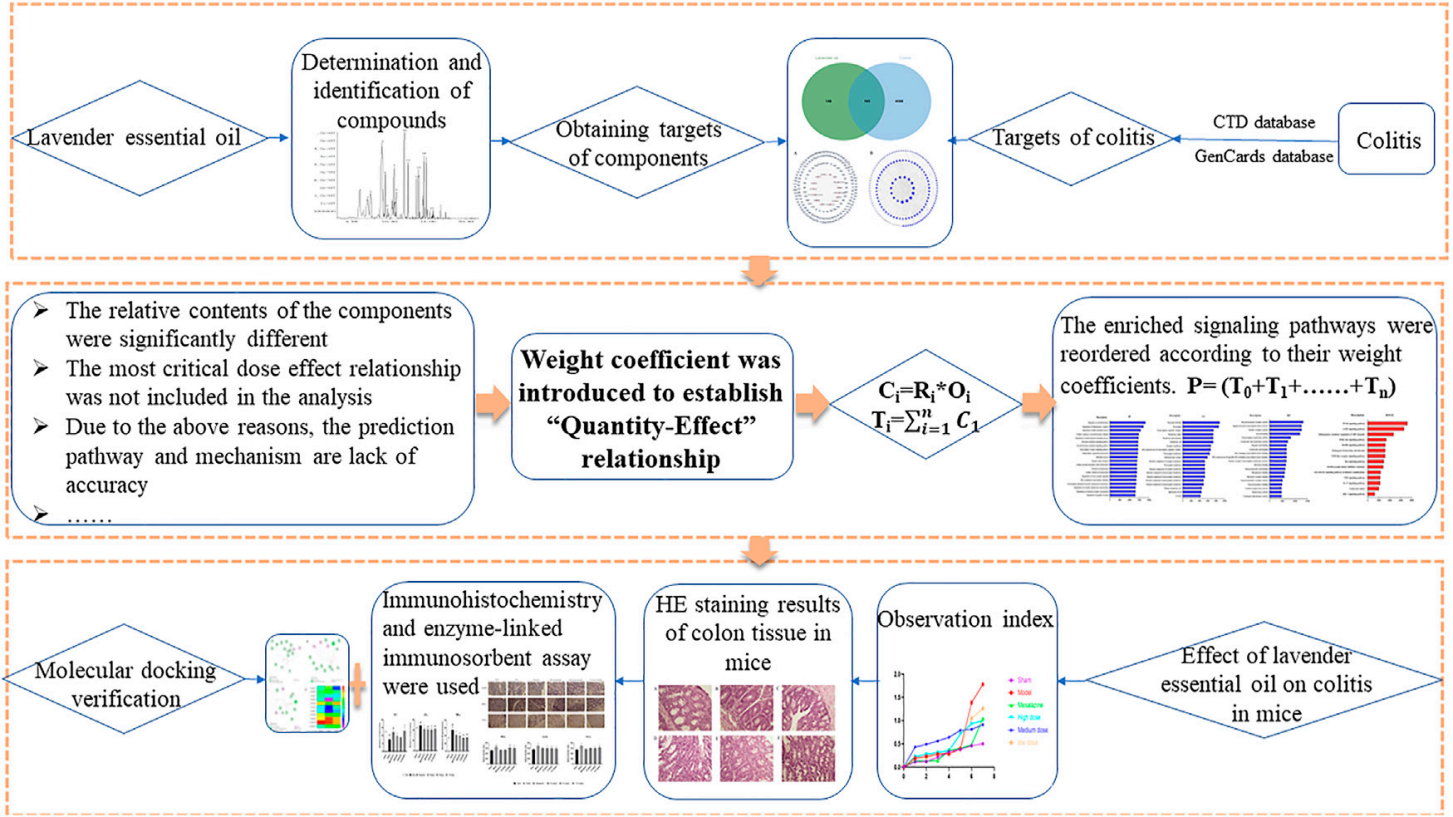
This article studies the flowchart.

Therefore, this study introduced lavender essential oil component content as a key factor, combined it with the OB value of each component, calculated its theoretical absorption into the blood content, and introduced a new weight coefficient combined with network pharmacology to predict the potential active components. Finally, we carried out a network pharmacology analysis, found the action targets and signal pathways of lavender essential oil, and investigated the therapeutic effect of lavender essential oil on colitis through animal experiments. The new “quantity–effect” weight coefficient network pharmacology can more accurately explore its mechanism of action and can provide a certain basis for more accurate and better application of network pharmacology. To further reveal the potential mechanism of lavender essential oil, this study used a multilevel and multiangle in-depth exploration strategy capitalizing on network pharmacology to provide a reference for further research and development of lavender essential oil for UC. The flow chart of this study is shown in Figure 1.

## Materials and Methods

### Isolation of Lavender Essential Oil Compounds

In this study, the chemical constituents of lavender essential oil were determined by gas chromatography–mass spectrometry (GC–MS). The gas chromatographic conditions used were as follows: Agilent HP-5 ms (30 m × 250 μ m × 0.25 μ m) capillary column, high-purity He, injection of 1.5 μL, no shunt, flow rate of 1 ml/min, programmed temperature of 65°C (retention time: 1 min), 5°C/min to 90°C (retention time: 1 min), and 10°C/min to 260°C (retention time: 10 min). The mass spectrometry conditions used were as follows: EI ion source, electron energy of 70 V, ion source temperature of 230°C, MS quadrupole temperature of 150°C, solvent delay of 3 min, mass spectrometry scanning mode of full scanning, and scanning range of 30–400 amu. The lavender essential oil was purchased from Xinjiang Ipalhan Spices Co., Ltd.

### Identification of Lavender Essential Oil Compounds

All data were processed by data analysis software, and the threshold was adjusted to 23 to screen the chemical composition of the lavender essential oil compounds. They were then searched in the NIST14 standard spectral database and screened according to the matching degree, the retention index, and the substances reported in the literature. The retention index was defined as the retention time of n-alkanes (C_8_–C_40_) under the same gas chromatographic conditions. According to the retention time of n-alkanes, the retention indices of volatile compounds from lavender essential oil were then calculated. The formula was as follows (Manral et al., 2008):

$$\text{RI}=\frac{100\text{n }+\;100\;\left[t_{R}\left(\text{x}\right)-t_{\text{R}}\;\left(\text{n}\right)\right]}{\left[t_{\text{R}}\;\left(\text{n }+\;1\right)-t_{R}\;\left(\text{n}\right)\right]},$$

where t_R_ (x), t_R_ (n), and t_R_ (*n* = 1) denote the retention time, carbon number n, and carbon number of n-alkanes to be tested and t_R_ (n) < t_R_ (x) < t_R_ (n = 1), respectively.

### Network Pharmacology Analysis Based on “Active Ingredient—Targets”

#### Acquisition of Targets for Lavender Essential Oil and Colitis-Related Diseases

All the identified active components of lavender essential oil were selected as target components. We then input the CAS number of each compound of lavender essential oil into the PubChem database (https://pubchem.ncbi.nlm.nih.gov/) to search based on its SMILES number. The number of smiles was further input and stored in the SwissTargetPrediction database (Gfeller et al., 2014; Zhang et al., 2020) (http://www.swisstargetprediction.ch/). The species "Homo sapiens" was selected to collect the target compounds of lavender essential oil. The related targets of colitis disease were obtained using the GeneCards databases (Amberger and Hamosh 2017; Marilyn et al., 2003) (https://www.genecards.org/). The keywords included “colitis” and “acute colitis.” The active compound prediction target of lavender essential oil and the prediction target of colitis-related diseases were imported into Bioinformatics & Evolutionary Genomics (http://bioinformatics.psb.ugent.be/webtools/Venn/) online software to draw a Venn map to determine the intersection, that is, the targets of lavender essential oil acting on colitis.

#### Construction of a Colitis Disease Target and Lavender Essential Oil Active Component Network

A network map of “component–target” was established using Cytoscape v3.7.1 (Shannon et al., 2003). The target data of lavender essential oil on colitis were then imported into Cytoscape v3.7.1. Using the “Network Analyzer” function in this software, the network topology properties were analyzed, and the important network topology parameters, including value and intermediate centrality, were investigated. Each node represented an active component and potential target of lavender essential oil and also showed the relationship between the component of lavender essential oil and its action targets.

#### Construction of a Target-Protein Interaction Network

String (https://string-db.org/) is a database for analyzing protein–protein interactions. We then uploaded the obtained targets to the string database, limited the research species to “homo sapiens,” set the lowest interaction score to 0.400, and hid the nodes that were not related to each other. Then, we obtained the protein interaction relationship, exported the data file, retained node 1, node 2, and the connection score data in the file, and imported them into Cytoscape v3.7.1 software. We then set parameters to make the node size and color depth reflect the value and the thickness of the edge to reflect the combination rate score. Then, we created a PPI network diagram.

### Establishment of Weight Coefficient

Lavender essential oil includes a variety of active ingredients, but the relative content of components is significantly different between different batches and manufacturers. Oral bioavailability (OB) is an important parameter for measuring the pharmacokinetic process and drug properties of drugs *in vivo*. To better demonstrate the important role of key active ingredients in a pharmacological mechanism, we associated the two, and the relationship was established:

$${\text{C}}_{\text{i}}={\text{R}}_{\text{i}}*{\text{O}}_{\text{i}},$$

where C_i_ represents the blood inflow of component I, R_i_ represents the relative content of component I in the total component, and O_i_ (OB) represents the probability of oral bioavailability of component I.

$${\text{T}}_{\text{i}}=\overset{n}{\underset{i=1}{\sum }}C_{1},$$

where T_i_ represents the sum of the corresponding targets of component I.

$$\text{P}=\;({\text{T}}_{\text{0}}{+\text{T}}_{\text{1}}+......{+\text{T}}_{\text{n}}(\text{,}$$

which is the sum of target scores participating in pathway P.

The component weight coefficient is the product of two elements, and we calculate the weight coefficient of all components according to the formula. Because the drug has the characteristic of multiple components corresponding to multiple targets, the weight coefficient of the cumulative target, namely, T_i_, is calculated according to the weight coefficient of the component. Moreover, because the target participates in the process in which the path plays a role, the weight coefficient of each path can be known and reordered according to the weight coefficient. It can be seen that this study established the “quantity–effect” relationship step by step from point to point.

### Enrichment Analysis of Lavender Essential Oil on Predictive Targets of Colitis

The clusterProfiler package in R was used to analyze the Kyoto Encyclopedia of Genes and Genomes (KEGG) signal pathway enrichment and Gene Ontology (GO) biological process enrichment of the target genes of lavender essential oil in the treatment of colitis and to analyze the main signal pathways and biological processes affected by lavender essential oil in the treatment of colitis. As described in *Establishment of Weight Coefficient*, the enriched signal pathways were reordered according to their weight coefficient, and the weight coefficient of each pathway was the sum of the weight coefficients of all the targets in a specific pathway.

### Molecular Docking

From the RCSB-PDB database (http://www.RCSB.org/), the 3D crystal structures of five key targets (PDB format): EGFR (PDB ID: 4lRM), MAPK3 (PDB ID: 6GES), and MAPK1 (PDB ID: 6G54) were obtained to remove the water molecules in the structure and hydrogenation. The key target and its corresponding components were docked. The 2D structure (SDF format) of the active components was then downloaded from PubChem. The position coordinates of the self-ligands in the protein were defined as the active pocket, that is, the docking site. Then, Discovery Studio Client 4.5 software was used for batch molecular docking analysis (Peng et al., 2018; YanJun and KaiCong 2019).

### Study of Lavender Essential Oil in the Treatment of Colitis in Mice

#### Experimental Animals

Sixty SPF male SD mice weighing (20 ± 2) g were purchased from Chengdu Dashuo Experimental Animal Co., Ltd., with animal license number SCXK (Sichuan) 202011. Under our standard experimental conditions, animals were fed adaptively for one week. This study was approved by the Animal Ethics Committee of Shaanxi University of Traditional Chinese Medicine.

#### Establishment of an Acute Colitis Model Induced by Dextran Sulfate Sodium in Mice

Sixty SPF male SD mice were randomly divided into 6 groups with 10 mice each. These included a blank group, a model group, a positive drug group, and lavender essential oil high- (50 mg kg^−1^ day^−1^), middle- (25 mg kg^−1^ day^−1^), and low-dose groups (12.5 mg kg^−1^ day^−1^). With the exception of the mice in the blank group, which were given distilled water, the mice in the other groups were free to drink a 3% dextran sulfate sodium (DSS) solution to induce a UC model, and the DSS solution was changed every other day (Hoffmann et al., 2018). At the same time, the mice in the positive drug group and lavender essential oil groups were given corresponding drugs, while the mice in the blank group and model group were given distilled water for 8 days.

#### Observation Indicators

Throughout the experiment, the general conditions of mice in each group were observed and recorded every day, including mental status, activity, fecal characteristics, and hematochezia, as well as changes in colonic lesions before and after treatment according to the disease activity index (DAI) score standard (Murano et al., 2000) (Table 1). This score is based on whether a water sample adheres to the anus (sparse stool), the paste stool, which does not adhere to the anus (semisparse stool), or a molded stool, which is present (normal stool). The calculation method was DAI = (body weight decline score + stool trait score + hematochezia score)/3.

**TABLE 1:** Disease activity index score.

Score	Body mass decline	Fecal state	Hematochezia
**0**	0	Normal	Negative (−)
**1**	0∼5	Between the two	Between the two
**2**	5∼10	Semithin stool	Occult blood (+)
**3**	10∼15	Between the two	Between the two
**4**	≥15	Sparse stool	Naked eye bloody stool

#### Sample Collection and Processing

All mice were euthanized after the last drug administration, and blood was collected. The serum was collected by centrifugation (4°C, 4000 r·min-1) for 10 min and stored at −80°C. After euthanasia, the colon of each mouse was dissected, and the distal colon was divided into 2 parts. One part was fixed with paraformaldehyde, while the other part was frozen in liquid nitrogen and transferred to a −80°C freezer.

#### Histopathological Determination

After fixation with 4% paraformaldehyde and embedding in paraffin, the colon tissue was cut into 5-μm sections for HE staining, and then, histopathological examination was performed using a light microscope (OLUMPUS, Japan) at 200X magnification.

#### Determination of EGFR (Epidermal Growth Factor Receptor), TNF-α (Tumor Necrosis Factor-α), and IFN-γ (Interferon-γ) by Enzyme-Linked Immunosorbent Assay (ELISA)

After centrifugation, the serum was extracted and the standard substance in the ELISA kit was diluted. We took the enzyme plate and marked the standard hole, blank hole, and sample hole. Then, we diluted the standard according to the instructions and then put the diluted sample into the sample hole. The antibody working solution, enzyme binding working solution, chromogenic solution, and termination solution were put in sequence. The absorbance was recorded at 450 nm of the spectrophotometer, and the standard concentration curve was made to calculate the content of EGFR, IFN-γ, and TNF-α in the sample.

#### Immunohistochemical Investigation of EGFR, IFN-γ, and TNF-α in Tissues

Colon tissue was sectioned at a thickness of 5 μm, soaked in xylene twice, and then hydrated for 10 min using an ethanol gradient (100%, 95%, 85%, 75%, and pure water). After antigen retrieval for 10 min in 3% H_2_O_2_, sections were incubated with nonimmunized, normal goat serum sealing solution (BSA) for 30 min, one antibody was added, and the wet box was maintained overnight at 4°C. The sections were then washed 3 times at room temperature for 40 min and incubated with HRP-labeled second antibody (rabbit anti) at 37°C. The slices were taken out, HRP-labeled avidin was added, and the slide was then incubated at 37°C for 30 min. The slices were next soaked in PBS for 3 min, which was repeated three times. The sections were then dyed with DAB chromogenic agent, rinsed in tap water, and re-dyed in hematoxylin. Next, the sections were immersed in 0.1% hydrochloric acid-ethanol to and were observed to control the staining degree under a microscope. After that, slides were dehydrated, mounted, and sealed. The staining was observed under a microscope and photographed with a 400X microscope. The integral absorbance of each slice was measured using a medical image analysis system, and the expression of EGFR, IFN-γ, and TNF-α protein was evaluated.

#### Statistical Analysis

All experimental data were statistically analyzed using SPSS 19.0 software, and the results are expressed as the mean ± the standard deviation (SD). One-way analysis of variance (ANOVA) was used to analyze the difference between the two groups. *p* < 0.05 was considered a statistically significant difference between the two groups.

## Results

### Active Ingredients of Lavender

**FIGURE 2:**
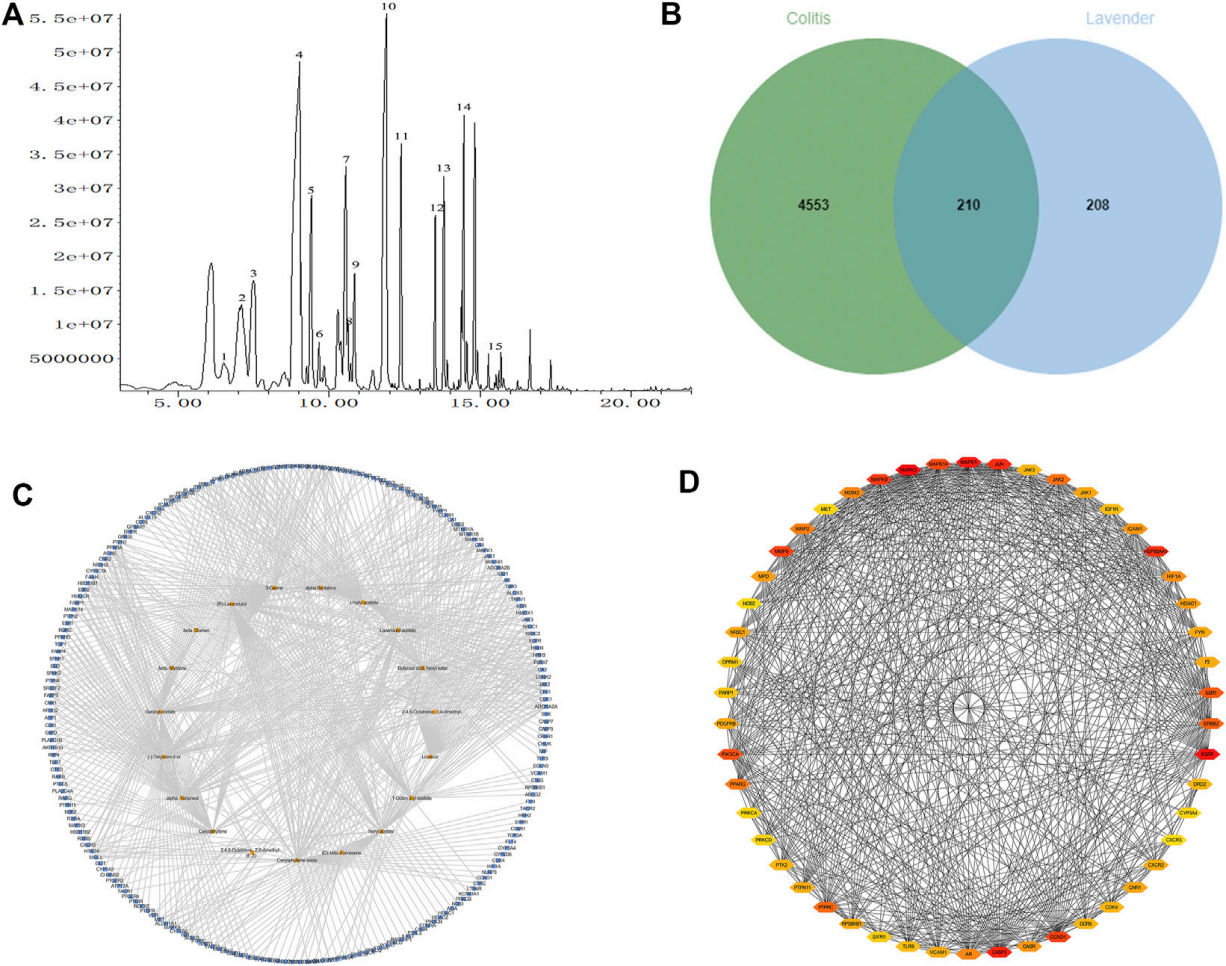
**(A)** Ion current diagram of lavender essential oil. **(B)** Active components of lavender essential oil and UC-related target Venny diagram. **(C)** Diagram of compound-disease target network. **(D)** PPI network and PPI network screening chart.

A total of 19 lavender essential oil compounds were resolved using GC-MS (Table 2). Figure 2A shows the ion mass spectrum of lavender essential oil.

**TABLE 2:** Chemical composition of lavender essential oil.

Number	Chemical compound	Retention index	Retention time (min)	Pct total
1	Beta-myrcene	996.5813	6.1048	9.642
2	3-Carene	1037.5394	7.1052	7.549
3	Beta-ocimene	1053.9201	7.5053	6.203
4	Linalool	1119.3528	9.0309	21.999
5	1-Octen-3-yl-acetate	1121.3650	9.0725	1.254
6	2,4,6-Octatriene, 3,4-dimethyl-	1137.9026	9.4144	4.91
7	2,4,6-Octatriene, 2,6-dimethyl-, (E,Z)-	1150.4014	9.6728	0.746
8	4-Hexen-1-ol, 5-methyl-2-(1-methylethenyl)-, (R)-	1180.6423	10.2980	2.198
9	3-Cyclohexen-1-ol, 4-methyl-1-(1-methylethyl)-, (R)-	1193.1459	10.5565	5.981
10	Butanoic acid, hexyl ester	1196.3674	10.6231	1.057
11	Alpha-terpineol	1208.1718	10.8399	2.426
12	Tricyclo [2.2.1.0 (2,6)]heptane, 1,3,3-trimethyl-	1269.7116	11.9070	16.095
13	4-Hexen-1-ol, 5-methyl-2-(1-methylethenyl)-, acetate	1297.5951	12.3905	4.334
14	2,6-Octadien-1-ol, 3,7-dimethyl-, acetate, (Z)-	1371.7777	13.5159	2.155
15	Geranyl acetate	1391.1047	13.8077	3.097
16	Tricyclo [2.2.1.0 (2,6)]heptane, 1,7-dimethyl-7-(4-methyl-3-pentenyl)-, (-)-	1433.1223	14.3912	0.995
17	Caryophyllene	1438.6489	14.4663	4.627
18	(E)-beta-farnesene	1465.0231	14.8247	3.94
19	Caryophyllene oxide	1608.6612	16.6587	0.792

### Prediction of Disease Targets of Colitis and Prediction of Related Targets of Lavender Essential Oil in the Treatment of Colitis

A total of 1476 targets of lavender essential oil were obtained using the SwissTargetPrediction database. After eliminating the duplicate values, 466 were left. Colitis-related targets were obtained from the GeneCards database, and a total of 4746 targets were included. A total of 210 targets were identified by considering the interaction between colitis-related targets and potential targets of lavender essential oil, as shown in Figure 2B.

### Prediction of Composition Targets and Protein Interaction Network Diagram of Lavender Essential Oil

The data of active components and colitis target of lavender essential oil were imported into Cytoscape v3.7.1 software to construct the “active components target” network of lavender essential oil in the treatment of colitis, and the network topology parameters were calculated by network analysis. In the output network, the active components, targets, and action pathways are represented by nodes with different colors, the orange square node represents the active components of lavender essential oil, and the blue square node represents the action targets, as shown in Figure 2C. The network consists of 228 nodes (19 compounds and 210 targets) and 725 connections.

### PPI (Protein Protein Interaction) Network Analysis

The PPI network consists of 50 nodes and 587 edges. The target points larger than the average degree value of 22.6 were selected to draw the network diagram. As shown in Figure 2D, the node size represents the degree value and the edge thickness represents the connection score. The larger the degree value is, the more critical it is. It may be the key target protein of lavender essential oil in the treatment of colitis. PPI network analysis showed that MAPK3 (degree: 100), EGFR (degree: 85), MAPK1 (degree: 84), CASP3 (degree: 80), and JUN (degree: 70) were the main targets of the lavender essential oil regulation pathway.

### Bioinformatic Analysis

**FIGURE 3:**
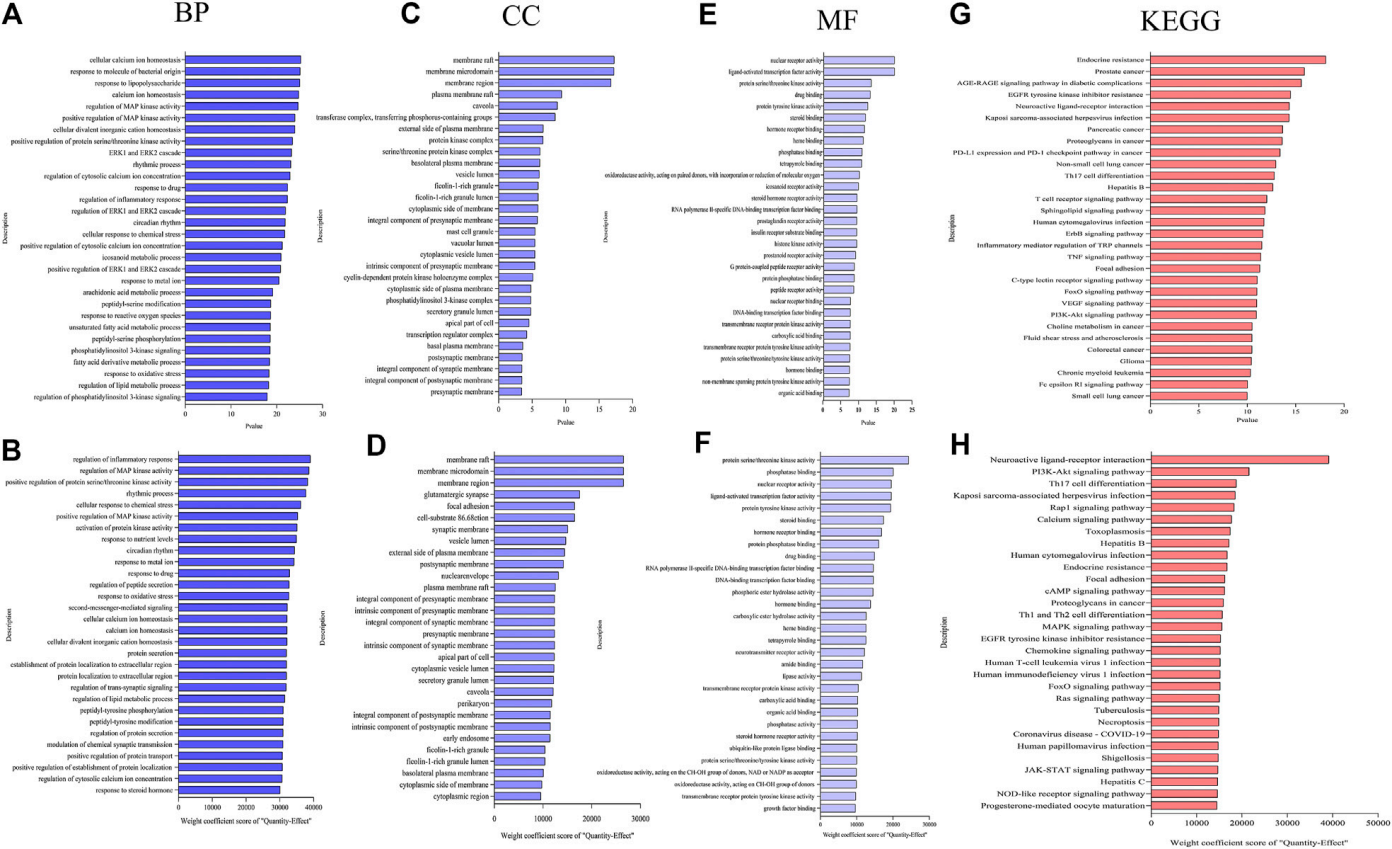
**(A–F)** Biological process (BP), cellular component (CC), and molecular function (MF) of lavender essential oil before and after weight coefficient calculation were analyzed. **(G, H)** KEGG enrichment analysis of target compounds in lavender essential oil before and after ranking by weight coefficient algorithm.

In this study, the target genes of lavender essential oil were enriched and analyzed by the cluster profiler package in R language of Rstudio software, and 2254 items of biological process (BP), 218 items of molecular function (MF), and 79 items of cellular component (CC) were obtained. According to item 2.4, the enriched items were reordered, and the top-30 histogram was drawn compare separately. As shown in Figure 3A, the targets before sorting were mainly concentrated in cellular calcium ion homeostasis, response to molecule of bacterial origin, and response to lipopolysaccharide, and as shown in Figure 3B, after sorting by weight coefficient, it focuses on regulation of inflammatory response, regulation of MAP kinase activity, positive regulation of protein serine/threonine kinase activity, and other biological processes, with obvious difference before and after sorting. Figures 3C, D show membrane raft, membrane microarea, membrane area, and other cell components. Before and after sorting, except for the first three ranking places, the other ranking differences are more significant. As shown in Figure 3E, the sequenced targets mainly focus on the molecular functions of protein serine/threonine kinase activity, phosphatase binding, and nuclear receptor activity. Before sorting, the target is mainly focused on the nuclear receptor activity, ligand-activated transcription factor activity, and protein serine/threonine kinase activity, as shown in Figure 3F. The difference between before and after sorting is small.

Next, 158 signaling pathways were screened by KEGG pathway enrichment analysis. According to item 2.4, they were reordered according to the weight coefficient, and the top 30 pathways were listed. The bubble chart was drawn, as shown in Figure 3G, and the main paths before sorting are endocrine resistance, promote cancer, and AGE-RAGE signaling pathway in diabetic complications. As shown in Figure 3H, the main pathways were the PI3K-Akt signaling pathway, Th17 cell differentiation, Th1 and Th2 cell differentiation, involving the neuroactive ligand-receptor interaction, Kaposi’s sarcoma-associated herpesvirus infection, and calcium signaling pathway. After sorting, the pathways involved before sorting were quite different from those involved in regulating colitis.

### Analysis of Molecular Docking Results

**FIGURE 4:**
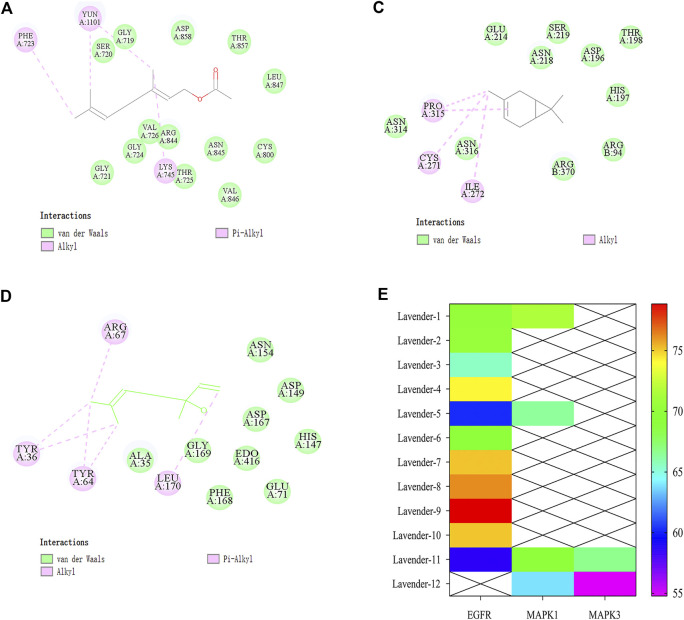
Docking of lavender active components with key targets.

The potential active components and key targets of lavender essential oil in the treatment of colitis were analyzed by molecular docking (Table 3). The results of this study showed that most of the docking scores of key targets with active components of lavender essential oil were higher than those with positive drugs, indicating that there was binding activity between compounds and target proteins, as shown in Figure 4D. And some 2D conformations with high scores were selected for display. The results are shown in Figures 4A-C. It is speculated that these components may play an important role in regulating the expression of colitis target proteins or reacting with these proteins.

**TABLE 3:** Molecular docking result of key components and corresponding key targets.

Number	Chemical compound
Lavender-1	Linalool
Lavender-2	1-Octen-3-yl-acetate
Lavender-3	4-Hexen-1-ol, 5-methyl-2-(1-methylethenyl)-, (R)-
Lavender-4	Butanoic acid, hexyl ester
Lavender-5	Alpha-terpineol
Lavender-6	Linalyl acetate
Lavender-7	4-Hexen-1-ol, 5-methyl-2-(1-methylethenyl)-, acetate
Lavender-8	2,6-Octadien-1-ol, 3,7-dimethyl-, acetate, (Z)-
Lavender-9	Geranyl acetate
Lavender-10	Caryophyllene oxide
Lavender-11	Mesalazine
Lavender-12	3-Carene

### Study on the Therapeutic Effects of Lavender Essential Oil on Colitis in Mice

#### Comparison of Disease Activity Index Scores

**FIGURE 5:**
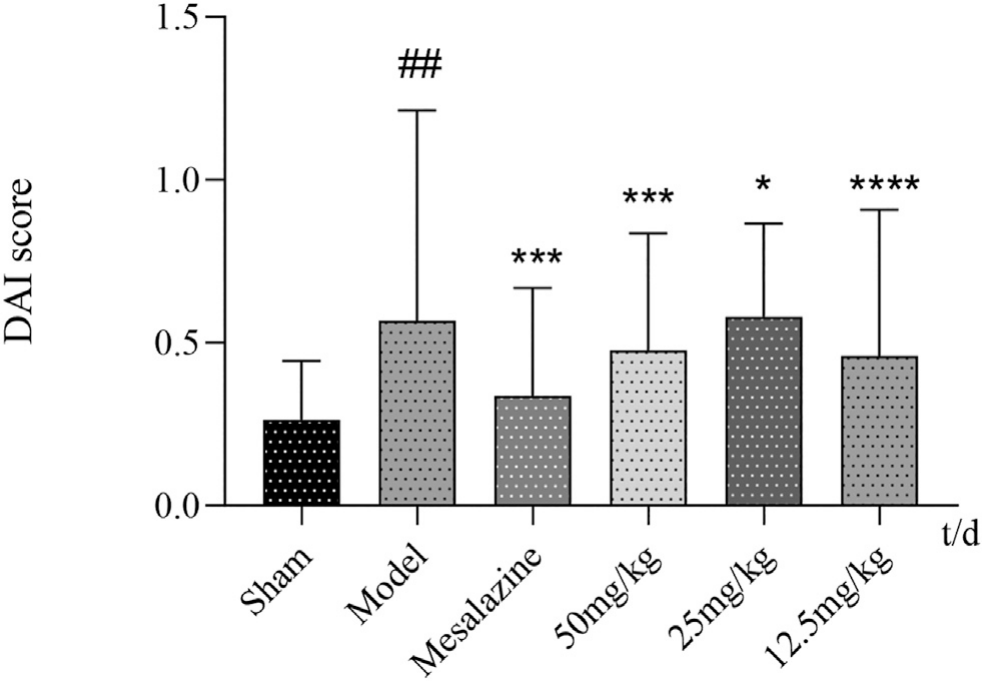
DAI score curve of mice in each group. ^#^ *p* < 0.05 vs. control group and *p < 0.05 vs. model group.

The mice in the blank group moved freely, had a good mental state, and continued to gain weight over the course of the experiment. The mice from other groups began to have watery stools starting on the second day of modeling, while some mice had stools mixed with mucus, and their food intake decreased; they started showing signs of lazy movement and arched backs. On the fourth day of modeling, they began to have mucus purulence, bloody stools, and stools mixed with blood. Moreover, their general state was poor, and their food intake and body weight had decreased. Over the course of the experiment, the body weight of the mice in the model control group did not increase significantly until the end of the experiment, and the mice still had mucus, purulence, and bloody or sparse stool. Their hair was also dull, thin, and slow in action. After drug intervention in each group, the body weight of the mice in each group began to increase after 4 days of drug intervention, as can be seen from Figure 5. On the first day of drug intervention, the mucus purulence and bloody and loose stool in mice were better than before, while after the 6th day, mice in each treatment group only had loose stool, with mucus-purulent blood being rarely seen. At the end of the experiment, there was no mucus purulence or bloody stool in each treatment group, and the number of mice with thinning stools decreased, especially in the lavender essential oil and mesalazine groups.

#### Morphology of Colonic Tissue From Mice After Treatment With Lavender Essential Oil Serum Expression of TNF-α, EGFR, and IFN-γ

Results in Figure 5 show that the contents of EGFR, TNF-α, and IFN-γ in the model group were significantly higher than those in the blank group (*p* < 0.05 and *p* < 0.01). Compared with the model group, the expression levels of serum TNF-α and IFN-γ in the mesalazine group and lavender essential oil high-, medium-, and low-dose groups were decreased in varying degrees (*p* < 0.05 and *p* < 0.01). Compared with the model group, the serum expression levels of the mesalazine group and lavender essential oil high- and medium-dose groups were significantly decreased (*p* < 0.01), while those of the low-dose group were significantly lower than those of the model group (*p* > 0.05).

#### Morphology of Colonic Tissue From Mice after Treatment With Lavender Essential Oils

The results of HE staining showed that the morphology of the intestinal mucosa in the normal group was normal and no obvious abnormalities were found. The number of goblet cells in the mucosal layer of the model group decreased sharply; however, a small amount of inflammatory cell infiltration could be seen in the tissue. Individual inflammatory cell infiltration could be seen in the intestinal mucosa of mice in the mesalazine and high-dose lavender essential oil groups. Moreover, the intestinal mucosal edema and inflammation of mice in the mesalazine and high-dose lavender essential oil groups were similar to those in the lavender essential oil high-dose group, as shown in Figure 7. In the middle- and low-dose lavender essential oil groups, the overall structure of the intestinal tissue was normal, the intestinal villi were arranged neatly, the epithelial cells of the mucous layer were not exfoliated or necrotic, the number of goblet cells of the mucous layer did not significantly decrease, and no obvious inflammatory cell infiltration was found in these tissues.

**FIGURE 6:**
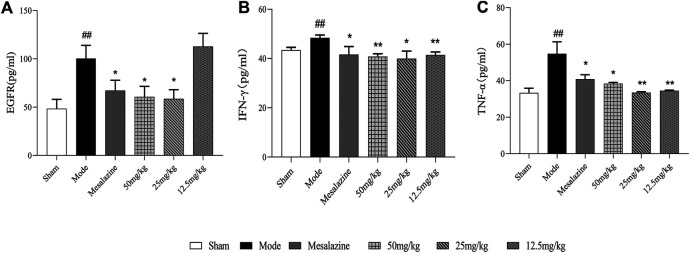
Effects of lavender essential oil on EGFR, IFN-γ, and TNF-α levels in serum of model mouse ^#^ *p* < 0.05 vs. control group and ^*^ *p* < 0.05 vs. model group.

**FIGURE 7:**
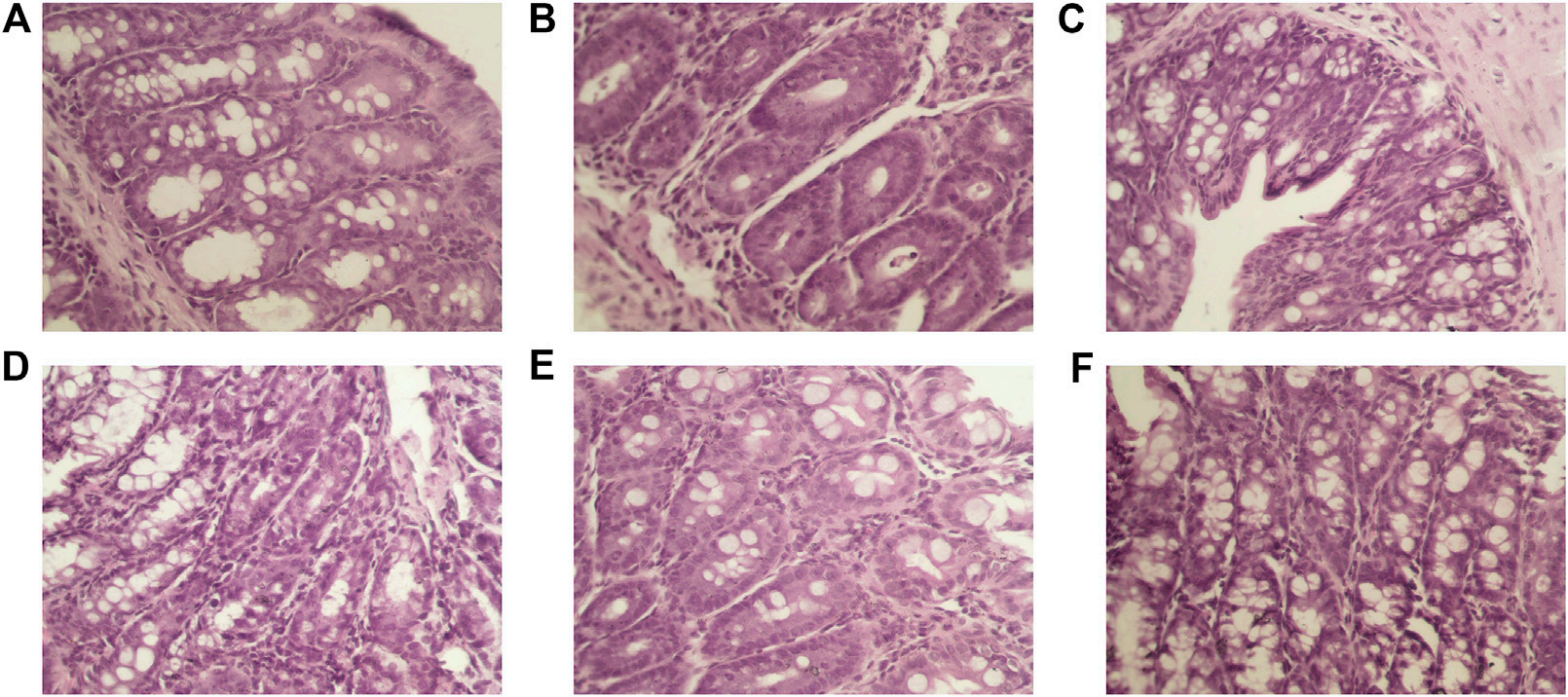
Effects of lavender essential oil on colonic pathological damage (×200).

#### Expression of EGFR, TNF-α, and IFN-γ in Colonic Mucosa

**FIGURE 8:**
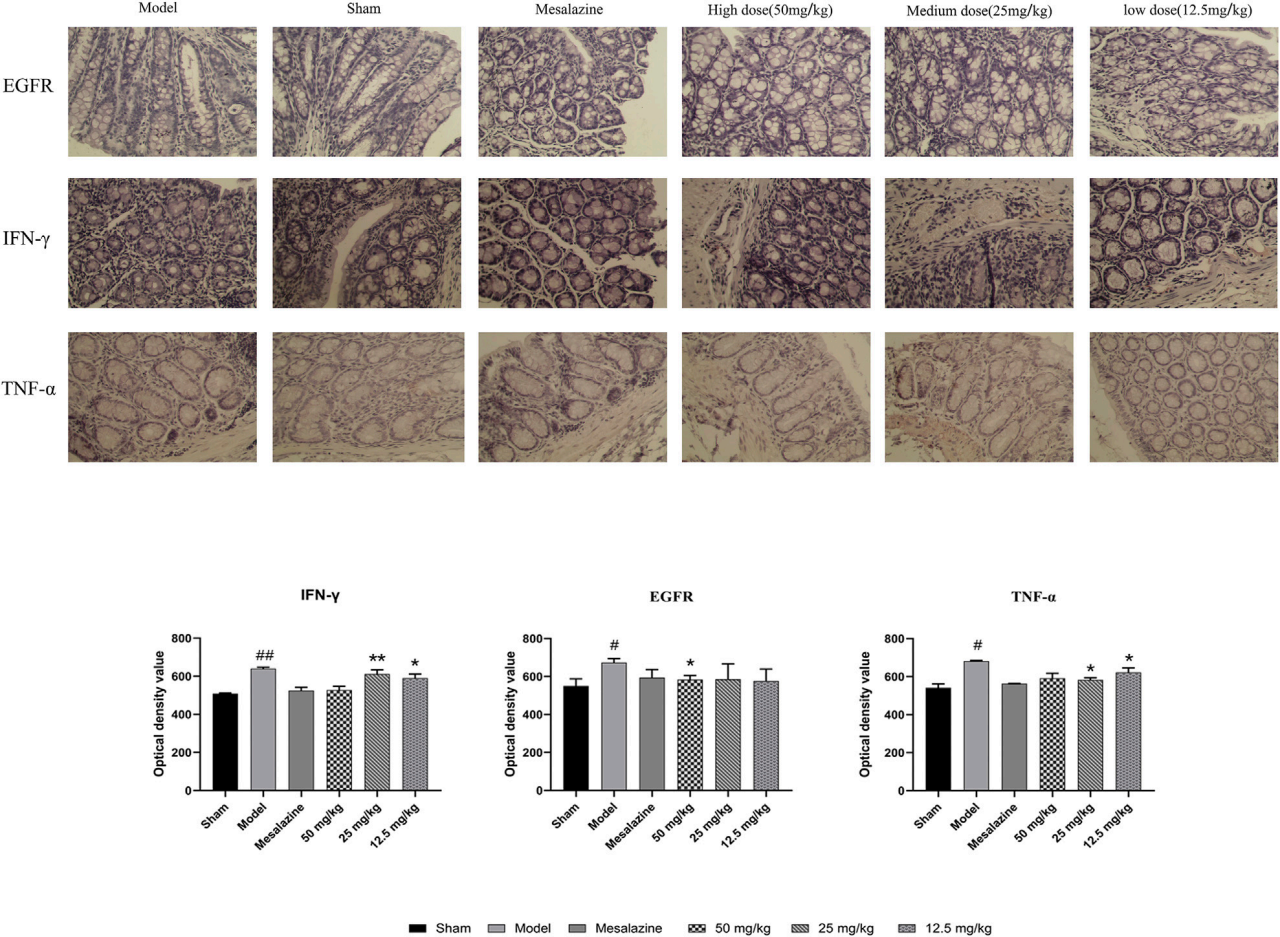
Effects of lavender essential oil on pathological changes and expression of EGFR, IFN-γ, and TNF-α in colonic tissue of model mouse (×200). Effects of lavender essential oil on expression of EGFR, IFN-γ, and TNF-α in colonic tissue of model mouse (Immunohistochemistry). ^#^ *p* < 0.05 vs. control group; ^*^ *p* < 0.05 vs. model group.

Compared with the control group, the expression levels of EGFR, TNF-α, and IFN-γ in the DSS group were significantly increased (*p* < 0.05). There was no significant difference in the protein expression of EGFR, TNF-α, and IFN-γ between the mesalazine group and lavender essential oil group (*p* > 0.05). Compared with the DSS model group, EGFR, TNF-α, and IFN-γ in each treatment group were significantly decreased (*p* < 0.05, *p* < 0.01). The results indicated that lavender essential oil can inhibit the expression of EGFR, TNF-α, and IFN-γ in colonic tissue of mice to inhibit its inflammatory activity, as shown in Figure 8.

## Discussion

Colitis was first reported over 160 years ago, but its pathogenesis is still under exploration as it is still unclear. In recent years, our understanding of colitis has deepened, and it is known that immune factors, genetic factors, and intestinal microorganisms are closely related to the occurrence of colitis (Kanauchi et al., 2003; Hisamatsu et al., 2013). An increasing number of studies have shown that the interaction between the immune system and intestinal microorganisms may be one of the most important drivers of the occurrence and protracted healing of colonic inflammation (Andoh et al., 2011).

In research on network pharmacology, the content of drug components is often ignored, but the influence of content and concentration on drug efficacy cannot be ignored. At present, OB is generally used as a screening condition to determine the effective components; that is, only the components that can be well absorbed after oral administration can become effective components, but the effect of component content on the efficacy is ignored (Gao 2018). In addition, there are many chemical components in the same medicinal material, and the proportion of each component is also different, so it is unreasonable to treat all the single components equally. The content and action of the active ingredients in medicinal materials are divided into primary and secondary, which cannot be regarded as the same. Although the content of some ingredients is low, it may be the key ingredient, while the content of some ingredients is high, but it may not work. Therefore, it is unreasonable to study the possible target and mechanism of action without considering the content of components and treating all components equally. The content of the “quantity–effect” relationship, which is the most critical to its efficacy, is not included in the analysis, resulting in some low-content components being predicted as key active components, which, in turn, results in a lack of accuracy in predicting pathways and mechanisms. In the study of network pharmacology, the content of medicinal components can be considered by the weighted method, and the pharmacokinetic parameters of drug components can be used as a reference. At the same time, the phenomenon of different efficacies caused by the different content of drugs should be studied experimentally to determine the relationship between content and efficacy, which should be considered in the study of network pharmacology (Meng and Tang 2020).

To better understand the pharmacological mechanism of lavender essential oil in the treatment of colitis, we introduced a new coefficient-weight coefficient. The weight coefficient is the product of the relative content of each component in lavender essential oil and the oral bioavailability of each in the human body. It needs to be emphasized here that the weight coefficient is not an absolute coefficient but a concept of relative content. The order of the weight coefficient of each component can be considered as the degree of participation of that component in a pharmacological mechanism, so the higher the weight coefficient, the more molecules are bound and the greater the binding affinity between the components. For example, the pathways of KEGG, BP, CC, and MF were significantly different according to the "quantity–effect" weight coefficient, and signaling pathways such as Th17 cell differentiation (from 11th to 3rd after sorting) and Th1 and Th2 cell differentiation (from 70th to 14th after sorting) were more similar. Therefore, this study combined network pharmacology, the weight coefficient, and bioinformatics to explore the concept of the driving force of UC.

KEGG enrichment analysis showed that the weight scores of Th17 cell differentiation were high and ranked at the top. Among them, Th17 cell differentiation has proved to play an important role in the abnormal immune response of IBD (Abdulahad et al., 2008). In a study (Abdulahad et al., 2008), Th17 cells were found in the intestinal mucosa of patients with IBD-secreted IFN-γ, a marker of Th1 cells. IFN-γ is an important factor in the process of inflammation and immune response. It has strong immunomodulatory effects and can activate and enhance immune cells (Pang et al., 2007), mediating related anti-inflammatory mechanisms and thereby enhancing the killing effect on pathogenic microorganisms and infected cells (Corpuz et al., 2016; Ma et al., 2016). Carol et al. (Carol et al., 1998) found that normal intestinal mucosal lymphocytes and epithelial cells could spontaneously secrete a certain amount of IFN-γ under physiological conditions, suggesting that IFN-γ plays a certain role in maintaining normal mucosal immunity. Moreover, after isolation and culture of CD4+ T cells from inflammatory bowel diseased intestinal tissue and normal intestinal tissue, the secretion of IFN-γ by CD4+ T cells in diseased intestinal tissue was significantly higher than that in normal intestinal tissue after stimulation with CD2 and CD28 antibodies (Kwon et al., 2010). As the main effector molecule secreted by Th17 cells, IL-17 can promote and amplify its pro-inflammatory effect by acting alone or in synergy with TNF-α (Friedrich et al., 2015). TNF-α can promote neutrophil aggregation in inflammatory or ulcerated areas of colonic mucosa, release the platelet-activating factor to promote thrombosis, cause disturbance of colonic mucosal microcirculation, and weaken the intestinal mucosal barrier function, resulting in mucosal injury and inflammatory cell infiltration (Rodríguez-Perlvárez et al., 2012). A study of animal inflammatory bowel disease showed that most of the Th17 cells were found in the ileum and colon of mice, and the contents of Th17-related cytokines IFN-γ and TNF-α were significantly increased (Flavio, Francesco, and Giovanni, 2008). EGFR is a receptor tyrosine kinase, which can mediate multiple signal transduction pathways. After binding with EGF, it activates p85 and P110 near the cell membrane and then catalyzes phosphoinositol diphosphate on the inner surface of the membrane to produce PI3P. As a second messenger, it activates Akt, thereby inhibiting the differentiation of Th17 cells. It has therapeutic effect on dextran sulfate sodium-induced ulcerative colitis in mice (Siregar, Halim, and Sitepu, 2015; Zhang and Zou, 2015). This is consistent with the findings of this paper.

Based on weight network pharmacology, KEGG signaling pathway reordering, and the hub gene, this study selected Th17 cell differentiation and considered the classical factors TNF-α, IFN-γ, and EGFR as the detection index. The results showed that lavender essential oil could regulate the differentiation of Th17 cells and mediate the interaction of multiple signaling pathways. It has a therapeutic effect on dextran-sulfate-sodium-induced colitis in mice. This proves the reliability of the weight network pharmacology analysis to a certain extent.

In conclusion, the weight coefficient and network pharmacology were introduced to predict the regulatory mechanism of lavender essential oil in the treatment of DSS-induced ulcerative colitis in mice to provide the basis for clinical medication.

## Conclusion

Because of the drawbacks of network pharmacology prediction (namely, the fact that the content of the dose-response relationship, which is the most critical for its efficacy, is not included in the analysis), some low content components are predicted as key active components, which causes the prediction pathway and mechanism to lack accuracy. To solve the above problems, in this study, we introduced the parameter of the “quantity–effect” weight coefficient and carried out statistical analysis on the target components to obtain the “quantity–effect” weight values of different targets. Then, we calculated the weighted values of BP, CC, MF, and KEGG pathways of R-language enrichment network pharmacology and finally reordered the importance of their pathways to explore the real significance of the pathway and mechanism of action. The results of animal experiments were consistent with the results of network pharmacology mining, which verified the reliability of network pharmacology network prediction based on the “quantity–effect” weight coefficient and confirmed the accuracy of lavender essential oil in the treatment of colitis.

## Data Availability

The raw data supporting the conclusions of this article will be made available by the authors, without undue reservation, to any qualified researcher.
